# Supplementary material for: Reconciling Mining with the Conservation of Cave Biodiversity: A Quantitative Baseline to Help Establish Conservation Priorities
Source: PLoS One. 2016 Dec 20;11(12):e0168348. doi: 10.1371/journal.pone.0168348 (PMC5173368; doi:10.1371/journal.pone.0168348)
Supplement: S1 Dataset — (ZIP) [file pone.0168348.s002.zip › Taxa/Serra Norte/SN_2013/N4WS/Lista N4WS-73.pdf]

CAVIDADE N4WS-0073

Classe	Ordem	Fam/Outros	Gên/Outros	Espécie	S-U	U-S	Zon.
Annelida	Oligochaeta			sp.	2		A
Arachnida	Acari	(Parasitengonina)		sp.1		X	P
Arachnida	Acari	Argasidae	*Ornithodoros*	sp.1	X	X	E/P/A
Arachnida	Acari	Macrochelidae		sp.2		X	P
Arachnida	Acari	Macronyssidae		sp.1	X		A
Arachnida	Acari	Rhagidiidae		sp.1		X	E/P
Arachnida	Amblypygi	Charinidae	*Charinus*	jovem		2	E/P
Arachnida	Amblypygi	Phrynidae	*Heterophrynus*	*longicornis*	1	1	E
Arachnida	Araneae	Caponiidae		jovem		X	P/A
Arachnida	Araneae	Corinnidae	*Creugas*	sp.1	1	1	P
Arachnida	Araneae	Drymusidae		jovem	X		P
Arachnida	Araneae	Filistatidae		sp.1		X	E
Arachnida	Araneae	Ochyroceratidae	*Ochyrocera*	sp.1	X	X	P/A
Arachnida	Araneae	Ochyroceratidae	*Speocera*	sp.1	X	X	P
Arachnida	Araneae	Ochyroceratidae		sp.n.1		X	P
Arachnida	Araneae	Pholcidae		jovem		X	P
Arachnida	Araneae	Prodidomidae		jovem		X	E/P
Arachnida	Araneae	Theraphosidae		jovem		2	E
Arachnida	Araneae	Theridiidae		jovem		X	E/A
Arachnida	Araneae	Theridiosomatidae	*Plato*	sp.1	X	X	E/P/A
Arachnida	Opiliones	Escadabiidae		sp.1	X	X	P/A
Arachnida	Opiliones	Stygnidae		jovem	1		P
Arachnida	Palpigradi	Eukoeneniidae	*Allokoenenia*	sp.1		X	P
Arachnida	Pseudoscorpiones	Bochicidae		sp.1	X		A
Arachnida	Pseudoscorpiones	Chernetidae	*Spelaeochernes*	sp.1	X	X	E/P
Arachnida	Pseudoscorpiones	Chthoniidae	*Pseudochthonius*	sp.1		X	P
Arachnida	Pseudoscorpiones	Chthoniidae	*Pseudochthonius*	sp.3		X	P
Arachnida	Schizomida	Hubbardiidae	aff. *Rowlandius*	sp.1		X	P
Chilopoda	Scolopendromorpha	Cryptopidae	*Cryptops*	sp.1		1	E
Chilopoda	Scolopendromorpha	Scolopocryptopidae	*Newportia*	sp.1	2	2	P
Chilopoda	Scutigeromorpha	Pselliodidae		jovem		3	E/P
Diplopoda	Polydesmida	Aphelidesmidae		sp.1		X	P
Diplopoda	Polydesmida	Fuhrmannodesmidae		sp.1		X	P
Diplopoda	Polydesmida	Pyrgodesmidae		sp.1	X		A
Diplopoda	Polydesmida	Tridontomidae		sp.1		X	P
Entognatha	Collembola	Cyphoderidae		sp.1	X		A
Entognatha	Collembola	Isotomidae		sp.1		X	P
Entognatha	Collembola	Paronellidae		sp.4	X	X	E/P/A
Entognatha	Diplura	Campodeidae		sp.1	X	X	E/P/A
Entognatha	Diplura	Japygidae		sp.1		X	P
Insecta	Blattodea	Polyphagidae		sp.1	5	3	P/A
Insecta	Coleoptera	Carabidae	*Gen.n.*	sp.17	X		P
Insecta	Coleoptera	Scarabaeidae		jovem	X		E
Insecta	Coleoptera	Staphylinidae	Pselaphinae	sp.9	X		P
Insecta	Coleoptera	Tenebrionidae		jovem	X	X	P/A
Insecta	Diptera	Psychodidae	*Sciopemyia*	*sordellii*	X	X	E/P/A
Insecta	Diptera	Sarcophagidae		jovem		X	P
Insecta	Heteroptera	Reduviidae	Reduviinae	jovem	12	1	P/A
Insecta	Heteroptera	Reduviidae		sp.2		1	

Insecta	Heteroptera	Schizopteridae	Schizopterinae	sp.1	X	X	E/P
Insecta	Homoptera	Cixiidae		sp.3	X	X	P
Insecta	Hymenoptera	Formicidae	*Acanthostichus*	sp.1		X	P
Insecta	Hymenoptera	Formicidae	*Nylanderia*	sp.1	X		P
Insecta	Hymenoptera	Formicidae	*Nylanderia*	sp.2		X	P
Insecta	Hymenoptera	Formicidae	*Nylanderia*	sp.3		X	E/P/A
Insecta	Hymenoptera	Formicidae	*Octostruma*	sp.1	X	X	P
Insecta	Hymenoptera	Formicidae	*Pachycondyla*	sp.1	X		P
Insecta	Hymenoptera	Formicidae	*Pheidole*	sp.3		X	E
Insecta	Isoptera	Termitidae	*Convexitermes*	sp.		X	P
Insecta	Lepidoptera	Gelechioidea		sp.3		X	E
Insecta	Lepidoptera	Noctuoidea		sp.1	2		E
Insecta	Orthoptera	Phalangopsidae	*Phalangopsis*	sp.	39	67	E/P/A
Insecta	Psocoptera	Epipsocidae	*Epipsocus*	sp.2		X	E
Insecta	Psocoptera	Psyllipsocidae		jovem	X	X	P/A
Insecta	Thysanoptera	Thripidae		sp.1		X	P
Insecta	Thysanura	Nicoletiidae		sp.1		X	E
Gastropoda	Pulmonata	Subulinidae	*Lamellaxis*	sp.	X	X	P
Malacostraca	Isopoda	Scleropactidae	*Circoniscus*	*buckupi*	X	X	P
Symphyla		Scutigerellidae	*Hanseniella*	sp.1		X	E
Amphibia	Anura	Dendrobatidae	*Epipedobates*	sp.	1		E
Amphibia	Anura	Strabomantidae	*Pristimantis*	cf. *fenestratus*	8		E
Mammalia	Chiroptera	Emballonuridae	*Peropteryx*	sp.	4	2	P/A
Mammalia	Chiroptera	Furipteridae	*Furipterus*	*horrens*		7	
